# Risk factors associated with low bone mineral density and childhood osteoporosis in a population undergoing skeletal growth: a cross-sectional analytic study

**DOI:** 10.3389/fendo.2025.1587985

**Published:** 2025-05-23

**Authors:** Berta Magallares, Dacia Cerdá, Jocelyn Betancourt, Gloria Fraga, HyeSang Park, Helena Codes-Méndez, Estefanía Quesada-Masachs, Mireia López-Corbeto, Montserrat Torrent, Ana Marín, Silvia Herrera, Ignasi Gich, Susana Boronat, Jordi Casademont, Héctor Corominas, Jorge Malouf

**Affiliations:** ^1^ Department of Rheumatology, Hospital de la Santa Creu i Sant Pau, Barcelona, Spain; ^2^ Department of Rheumatology, Universitari Dexeus-Grupo Quirón Salud Hospital, Barcelona, Spain; ^3^ Institut de Recerca Sant Pau (IR SANT PAU), Barcelona, Spain; ^4^ Department of Rheumatology, Hospital Sant Joan Despí Moisès Broggi, Barcelona, Spain; ^5^ Department of Pediatrics, Hospital de la Santa Creu i Sant Pau, Barcelona, Spain; ^6^ Department of Pediatric Rheumatology, Vall d’Hebrón Barcelona Hospital Campus, Barcelona, Spain; ^7^ Department of Mineral Metabolism Unit - Internal Medicine, Hospital de la Santa Creu i Sant Pau, Barcelona, Spain; ^8^ Centro de Investigación Biomédica en Red de Enfermedades Raras (CIBERER), Madrid, Spain; ^9^ Department of Clinical Epidemiology and Public Health, Hospital de la Santa Creu i Sant Pau, Barcelona, Spain; ^10^ Department of Internal Medicine, Hospital de la Santa Creu i Sant Pau, Barcelona, Spain

**Keywords:** low bone mass for chronological age, childhood osteoporosis, bone fragility, bone mineral density, DXA (dual-energy x-ray absorptiometry)

## Abstract

**Background:**

Early identification of risk factors for low bone mass for chronological age (LBMca) and childhood osteoporosis (cOP) in patients undergoing skeletal growth is essential to mitigate long-term skeletal complications. cOP is diagnosed when LBMca (BMD Z-score ≤2) is accompanied by a clinically significant fracture history, or when vertebral fragility fractures are present.

**Methods:**

Patients under 21 years of age with at least one risk factor for LBMca (malabsorption syndrome, chronic inflammatory diseases, hematological diseases, endocrinopathies, drugs that affect bone metabolism, or insufficient calcium intake) were included. Data on fractures history and physical activity levels were collected. Spine and whole-body dual-energy x-ray absorptiometry (DXA) and vertebral morphometry were performed. Age-adjusted linear regression analysis evaluated associations between bone mineral density (BMD) and risk factors.

**Results:**

A total of 103 patients were included (mean age 9.8 years; 52.4% female), and 96.1% had more than two risk factors. The prevalence of LBMca was 10.5% and the prevalence of cOP was 4.8%. Vertebral BMD was positively associated with male sex. Whole body BMD was negatively associated with sedentary lifestyle and fracture history. Total body less head BMD showed negative associations with current steroid treatment, sedentary lifestyle, and history of fractures.

**Conclusions:**

Pediatric populations at risk of LBMca or cOP often have multiple risk factors, notably modifying ones such as physical inactivity. Up to 10.5% of children with risk factors present LBMca and 4.8% have an undiagnosed or unknown cOP. Longitudinal studies are warranted to understand the long-term impact of the identified risk factors, including age, sex, sedentary lifestyle, ethnicity and vitamin D status, on bone health.

## 1 Introduction

Currently, osteoporosis is a prevalent condition that may cause fractures due to bone fragility (1). Although these fractures mainly occur in individuals older than 50, the causes are often present during bone development at early ages (2). Reaching an adequate peak bone mass during adolescence is essential to prevent osteoporosis at older ages due to the effects of hormonal deficiencies and other factors associated with aging such as sarcopenia (3, 4).

Despite its clinical significance, Childhood Osteoporosis (cOP) remains frequently underdiagnosed in everyday pediatric practice. Its subtle presentation, lack of standardized screening protocols, and limited awareness among healthcare providers often delay recognition. As a result, many children at risk -particularly those with chronic conditions or on prolonged exposure to bone-impacting medications- may go unnoticed until fractures occur.

At present, the impact each osteoporosis-related risk factor has on bone development and their prevalence are unknown. Additionally, the short-term and long-term consequences of the risk factors present during childhood are unknown. The currently accepted primary risk factors for developing low bone mass for chronological age (LBMca) include insufficient calcium and vitamin D intake (5), sedentary lifestyle (5, 6) diseases that cause chronic inflammation (7), hypercalciuria (5, 6) and malabsorptive diseases (8). Drugs that affect bone metabolism, such as glucocorticoids or immunosuppressants, are also described as common causes of secondary osteoporosis (6, 9, 10).

In addition to the aforementioned risk factors, related conditions such as sarcopenia, a component of malnutrition, can adversely impact overall bone health and physical function by reducing skeletal muscle mass and muscle function, highlighting the importance of early detection and management through assessments of muscle mass, strength, and physical performance (11, 12).

The early detection of these risk factors may allow for better control of LBMca and treatment of cOP at early ages. Adherence to treatment is vital for effectively managing these conditions (13), as it helps prevent complications such as fractures and enhances overall patient care.

Since most of our knowledge regarding LBMca and Bone mineral density (BMD) was ascertained from adult populations, the objective of this study is to determine the risk factors for LBMca and BMD, and to describe the prevalence of risk factors for LBMca in a cohort of patients undergoing skeletal growth.

## 2 Materials and methods

We conducted a cross-sectional ambispective analytic study. Eligible patients were consecutively recruited from our Pediatric Rheumatology Outpatient Clinic between January 2018 and December 2020. Patients and/or their legal guardians gave their informed consent prior to recruitment. The study obtained approval from the institutional ethics committee of Hospital Sant Pau (IIBSP-FRA-2016-11). The study was conducted in accordance with the Helsinki Declaration.

### 2.1 Study population

Inclusion criteria were patients under 21 years of age who presented with at least one of the following risk factors: malabsorption syndrome, chronic inflammatory diseases, hematological diseases, endocrinopathies, treatment with drugs that affect bone metabolism (glucocorticosteroids or immunosuppressant drugs), or insufficient calcium intake. Patients who had previously received any anti-osteoporotic drugs were excluded.

Patients were classified as LBMca and cOP according to the International Society for Clinical Densitometry 2013 Pediatric Position Development Conference (14). The study was approved by the ethics committee at our hospital (IIBSP-FRA-2016-11). Informed consent was obtained from all patients and/or their legal guardians prior to recruitment.

### 2.2 Data collection and study variables

Electronic medical records from the hospital were used for data collection. All patients attended a baseline visit for a clinical interview and physical examination. Demographic and clinical variables were collected during the clinical interview. The patients who fulfilled the inclusion criteria underwent a dual-energy x-ray absorptiometry (DXA) and blood test. The following demographic and clinical variables were collected: age, sex, weight, height, disease history, history of previous fractures and current and past medication. Average calcium intake (milligrams/day) was calculated with the INDICAD 2001 study test (15), a validated, non-invasive questionnaire assessing dietary calcium consumption. Although we acknowledge potential recall bias inherent to dietary questionnaires, this method was selected for its feasibility and established use in routine clinical practice, allowing for consistent data collection across a wide age range. Physical activity was measured by the PAQ-A (Physical Activity Questionnaire for Adolescents) for patients older than 12. For patients younger than 12, the PAQ-C (Physical Activity Questionnaire for Children) was used. Both questionnaires were validated in the Spanish population (16, 17). Scores ranged from 1 (very low level of physical activity) to 5 (high level of physical activity). Data on physical activity were not collected for children under three years of age since the questionnaires are not validated for this age range.

Fasting laboratory parameters collected were calcemia, phosphatemia, OH-25-vitamin D concentration (determined by liquid chromatography coupled with tandem mass spectrometry), and calciuria from 6-hour urine collection. Outlier results from the 6-hour urine test were double-checked with a 24-hour calciuria test.

The following data were obtained by DXA: total body BMD, total body less head BMD, BMD at vertebrae L1-L4, and total body and vertebrae L1-L4 Z-score. Height adjustment for vertebral and total body Z-score values was performed for all cases by means of the formulas published by Zemel et al. (18). Densitometric determinations were obtained using an Hologic Discovery densitometer scanner (Hologic Discovery, Inc., Bedford, MA, USA) equipped with TBS iNsight^®^ software (Medimaps Group, Mérignac, France), and calibrated for pediatric use. All DXA scans were performed within one week of the clinical evaluation to ensure consistency between clinical and densitometric data.

The presence or absence of vertebral fractures was analyzed with vertebral morphometry by applying Genant’s semi-quantitative scale (19, 20).

### 2.3 Statistical analysis

Statistical analyses were performed with the IBM-SPSS (V26.0) software package. Quantitative variables are presented as mean (standard deviation). Categorical variables are presented as absolute frequencies and percentages. The relationship between categorical variables was assessed with contingency tables and the Chi square test, or Fisher’s exact test. The T-test was used to evaluate quantitative variables in comparison to a two-grouped categorical data analysis, and an analysis of variance was used in the case of more than two groups. The Mann–Whitney U test was used for non-normally distributed ordinal or quantitative variables in the case of two groups, and the Kruskal–Wallis test was used for more than two groups. Pearson’s linear correlation coefficient was used to correlate two quantitative variables. Spearman’s correlation coefficient was calculated when one of these variables or both were ordinal or showed a non-normal distribution in the Shapiro-Wilk test. Multivariable regression analyses were conducted adjusting for age and sex. However, due to the limited sample size and heterogeneity of the sample, a sensitivity analysis could not be performed. Potential cofounding factors were evaluated in the univariate analysis, stratified by risk factors. 95% confidence intervals were calculated for clinically relevant results. For all cases, the type I error level was 5% (α = 0.05) and a bilateral approximation was used.

An empirical sample size calculation was conducted based on an expected prevalence of LBM between 10% and 20%, with the inclusion of at least two key covariates (age and sex). Following the rule of one predictor per ten outcome events for multivariable analysis, a target sample size of approximately 100 participants was estimated. A *post-hoc* power calculation was then performed using the observed data. Assuming a small-to-medium effect size (f² = 0.10), two predictors (age and sedentary lifestyle), α = 0.05, and a desired power of 0.80, the required sample size was estimated at 100 participants. Our final cohort of 103 meets this threshold, providing an actual power of 0.803 and supporting the strength and stability of the planned analyses.

## 3 Results

### 3.1 Baseline characteristics and prevalence of the risk factors

A total of 103 patients were included for analysis, with a variety of comorbid diseases reflecting the diverse clinical backgrounds of the study population. The baseline characteristics of the population and the risk factors for LBMca are summarized in **Table 1**. Mean age was 9.8 years (SD ±4.7, range 2-20). The comorbidities included malabsorption syndromes and food allergies (mostly cow’s milk protein allergy and celiac disease, but also multiple food allergies, eosinophilic esophagitis, Chron’s disease, and short bowel syndrome), juvenile idiopathic arthritis (JIA) with its various subtypes (oligoarticular, polyarticular, enthesitis-related, psoriatic arthritis, and systemic JIA), nephropathies (including nephrotic syndrome, renal tubular acidosis, and chronic renal failure), hematological diseases (such as lymphoma, acute lymphoblastic leukemia, and graft-versus-host disease), systemic autoimmune diseases (including vasculitis, systemic lupus erythematosus, autoimmune hepatitis, and eosinophilic fasciitis), and autoinflammatory diseases (such as ADA2 deficiency, familial Mediterranean fever, and PFAPA syndrome).

**Table 1:** Baseline characteristics.

Variable	n (%)
Sex, female	54 (52.4)
Age (years; range)	
Early childhood (2-3)	9 (8.7)
Childhood (4-9)	33 (32)
Adolescence (10-17)	55 (53.4)
Young adulthood (18-20)	6 (5.8)
Ethnicity	
Caucasian	82 (79.6)
Other	21 (20.38)
Anthropometric characteristics	
Height ≤ 3^rd^ and 97^th^ percentile	7 (6.8) and 5 (4.9)
Weight ≤ 3^rd^ and 97^th^ percentile	9 (8.7) and 9 (8.7)
Number of Fractures (n) by patient	
None	85 (82.5)
1 fracture; long bone or vertebral	12 (11.7); 8
2 fractures; long bone or vertebral	4 (3.9); 4
≥3 fractures; long bone or vertebral	2 (1.9); 6
Comorbid disease	99 (96.1)
Malabsorption/food allergies	47 (46.6)
Juvenile idiopathic arthritis	18 (17.5)
Nephropathies	18 (17.5)
Hematological diseases	7 (6.8)
SARDs and autoinflammatory diseases	11 (10.67)
Endocrinopathies (pituitary hypoplasia)	1 (1)
Osteoporosis inducing medication	
Prior use of corticosteroid	40 (28.8)
Current use of corticosteroid	20 (19.4)
Other IS or chemotherapy	23 (22.33)
Other risk factors	
Insufficient dietary calcium intake	87 (84.5)
Sedentary lifestyle (PAQ<2)	14 (13.6)
History of long bone or vertebral fractures	13 (12.6)
Hypovitaminosis D in blood (<30nmol/L)	12 (11.7)
Hypercalciuria in 24-hour urine	4 (3.9)
N° Risk Factors	
1 risk factor	4 (3.9)
2 risk factors	40 (38.8)
3 risk factors	32 (31.1)
4 risk factors	15 (14.6)
5 risk factors	12 (11.7)

SARDs, systemic autoimmune rheumatic diseases; IS, immunosuppressants.

No patients were diagnosed with hypogonadism, delayed developmental milestones, or non-ambulatory conditions that could affect bone mass acquisition. This is important to note, as mechanical load related to gravity is essential for normal bone development, as is age (21).

At some timepoint, 40 patients (38.8% of the sample) had received systemic corticosteroid treatment, and 20 (19.4%) were receiving it at the time of recruitment. The mean daily doses and accumulated doses of prednisone in patients in current use were 6.9 mg/day (range 1.125 mg – 40 mg) and 8605 mg (median of 8283 mg), respectively. The mean accumulated dose of prednisone in patients with a prior use of corticosteroids was 4853 mg (median 2305 mg).

Data regarding daily calcium intake and daily recommended amounts (DRA) (22) are summarized in **Table 2**. Daily average calcium intake in diet was 696 mg. A decrease in adherence to calcium DRA was observed in accordance with age increase (p= 0.035). Median physical activity in each age group measured by PAQ was: 3.19 (IQ 25-75%:0.57) out of a maximum of 5 in school age subjects (4–9 years), 2.8 (IQ 25-75%:0.95) in adolescents (10-17years) and 2.45 (IQ 25-75%:1.67) in young adults.

**Table 2:** Daily mean calcium intake.

Age Group	DRA* (mg/day)	Mean Intake (mg/day)	SD	Range: min- max (mg/d)	% Meeting DRA
Pre-school (2-3y)	700	823	263	513-1346	44.4%
School (4-9y)	1000	655	233	254 - 1186	24.2%
Adolescent (10-17y)	1300	695	329	99 - 1925	10.9%
Young adult (18-20y)	1100	725	156	555 - 985	0%

DRA, daily recommended amount; SD, standard deviation.

For 100% of the cohort, calcemia was normal with an average of 2.49 (0.75) mmol/L. For 86% of the cohort, phosphatemia levels were normal with a mean of 1.57 (0.21) mmol/L, while 14% of patients presented elevated levels of serum phosphorus with a mean of 1.84 (0.16) nmol/L. The mean level of serum calcidiol was 66.82 (33.65) nmol/L. Calcidiol concentration was normal (≥ 30 nmol/L) [17] for 88% of measurements and deficient for 12%, with an average of 22.8 (3.9) nmol/L.

6-hour urine calciuria was obtained for 94% of the cohort. The concentration of calcium in urine was reduced (<1.6mmol/L) in 37.1% of the cohort (according to our local reference values), normal in 58.8% of the cohort, and high (> 5.3 mmol/L) in 4.1%, with a mean calciuria of 2.86 (1.1) mmol/L, 0.86 (0.4) nmol/L, and 7.65 (1.8) nmol/L, respectively.

### 3.2 Comparison of risk factors between patient groups

While statistically significant differences were observed when comparing the number of risk factors among the different comorbid disease groups (p<0.001), these findings should be interpreted cautiously due to small sample sizes. Patients with hematologic diseases presented the highest number of risk factors (5.4 ± 1.64), followed by those with systemic autoimmune diseases (3.75 ± 0.95), while patients with digestive diseases had the lowest number of risk factors (2.29 ± 0.7). A similar consideration applies to the observed proportion of fractures (57.1%) and sedentary behavior (71.4%) in the hematologic group.

When comparing risk factors between age groups, higher proportions of immunosuppressant treatment (43.6%, p=0.016) and lower daily calcium intake (p=0.035) were found in adolescents, followed by young adults. In addition, the number of risk factors increased with age (1.6 in pre-school children, 3.4 in adolescents, and 3.2 in young adults) (p<0.001). Sedentary lifestyle was most frequent among young adults (33.3%, p=0.017).

### 3.3 Densitometric results of the population

**Tables 3** and **4** show BMD values for the major body regions of interest by sex and the mean BMD for each comorbid disease. A higher age-adjusted vertebral BMD was observed in females (p=0.005), but it was not observed in total body BMD or in total body less head BMD (p=0.762 and p=0.902, respectively).

**Table 3:** BMD values of major body regions of interest by sex.

Sex	Bone Mineral Density				
	Total Body	Total Body Less Head	Vertebral	Femur (Total)	Femoral Neck
Female					
Mean (SD)	0.83 (0.16)	0.71 (0.16)	0.69 (0.18)	0.74 (0.16)	0.69 (0.15)
Range	0.54 – 1.17	0.41 – 1.01	0.39 – 1.06	0.44 – 1.17	0.40 – 10.30
Male					
Mean (SD)	0.79 (0.16)	0.66 (0.18)	0.59 (0.16)	0.74 (0.17)	0.68 (0.14)
Range	0.53 – 1.12	0.39 - 1	0.36 – 0.99	0.46 – 1.06	0.38 – 0.94

BMD, bone mineral density.

**Table 4:** Age-adjusted BMD of major body regions of interest by comorbid disease.

Comorbid disease	Bone Mineral Density		
	Vertebral Mean (SD)	Total Body Mean (SD)	Total Body Less Head Mean (SD)
Juvenile idiopathic arthritis (JIA)	0.70 (0.16)	0.86 (0.13)	0.73 (0.12)
Autoinflammatory diseases	0.76 (0.30)	0.94 (0.24)	0.78 (0.23)
Vasculitis	0.74 (0.17)	0.91 (0.14)	0.80 (0.14)
Connective tissue diseases	0.83 (0.23)	0.93 (0.18)	0.82 (0.17)
Malabsorption/food allergies	0.66 (0.17)	0.84 (0.14)	0.72 (0.14)
Hematologic diseases	0.69 (0.16)	0.84 (0.16)	0.76 (0.15)
Endocrinopathy	0.68 (0.13)	0.87 (0.11)	0.71 (0.19)

Results from subgroup analyses are exploratory. Limited sample sizes within diagnostic groups may affect the precision and generalizability of prevalence estimates.

BMD, bone mineral density; SD, standard deviation.

**Table 5** shows the proportion of LBMca in each comorbid disease group according to non-adjusted and height adjusted vertebral and total body Z-score.

**Table 5:** Proportion of LBMca by diagnosis groups before and after height adjustment.

Comorbid disease	Low Bone Mass for chronological age			
	Vertebral Z-score (%)	Vertebral Adjusted Z-score (%)	Total Body Z-score (%)	Total Body Adjusted Z-score (%)
Juvenile idiopathic arthritis (JIA)	11.1	5.6	11.8	0
Autoinflammatory diseases	0	0	0	0
Vasculitis	0	0	25	0
Connective tissue diseases	0	0	0	0
Malabsorption/food allergies	9.1	7.1	7	9.8
Hematologic diseases	28.6	33.3	50	60
Nephropathies	0	0	5.6	0
Total	8.2	6.4	10.5	7.7

Results from subgroup analyses are exploratory. Limited sample sizes within diagnostic groups may affect the precision and generalizability of prevalence estimates.

LBMca, low bone mass for chronological age (Z-score ≤ -2SD); Adjusted Z-score: height-adjusted Z-score.

**Figure 1:**
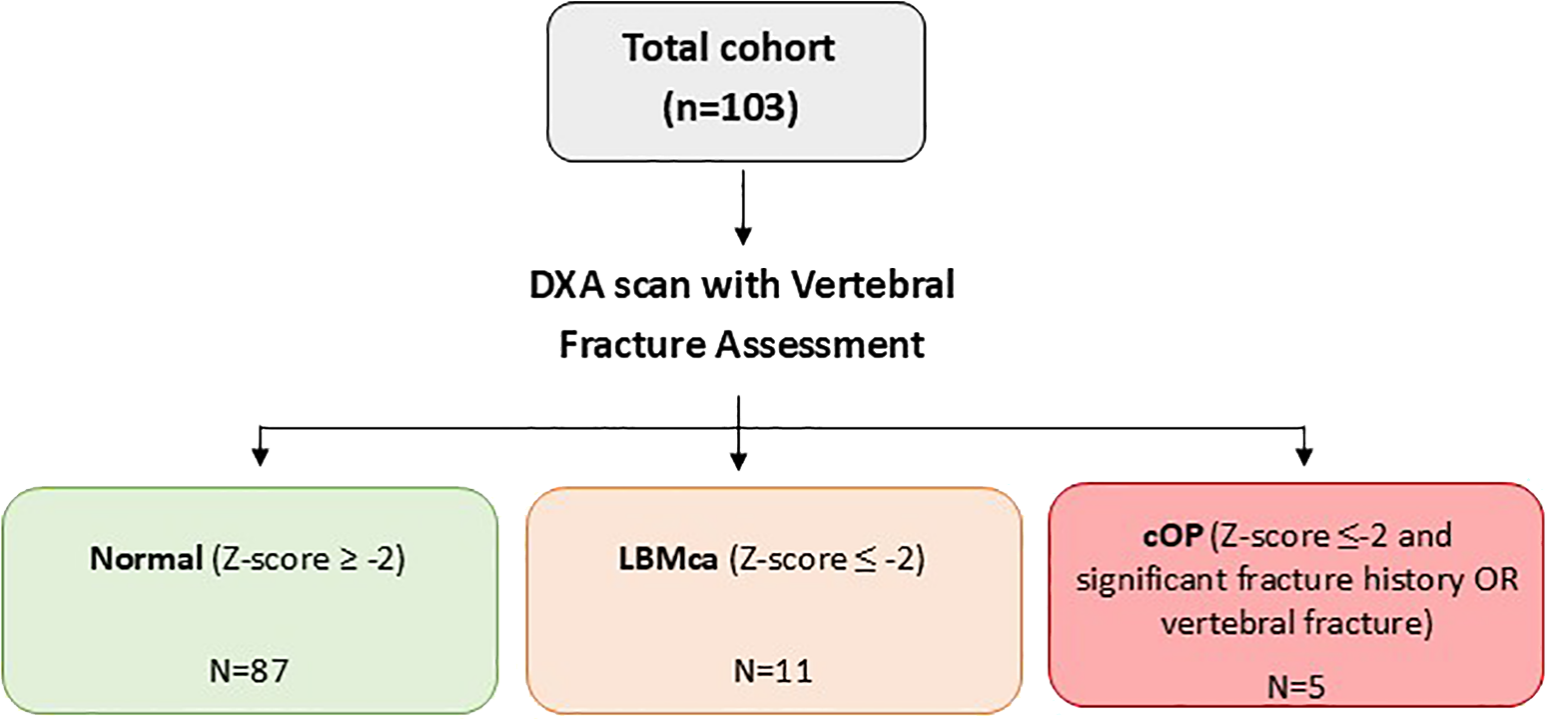
Flowchart study of population classification according to bone mineral density and fracture history. DXA, dual-energy x-ray absorptiometry; LBMca, Low Bone Mass for chronological age; cOP, Childhood Osteoporosis.

**Figure 1** shows the classification flow of the 103 patients included in the study according to their BMD Z-scores and fracture history. Of the total cohort, 11 patients met the criteria for LBMca, defined as a BMD Z-score ≤ -2. Among these, 5 patients also presented a clinically significant fracture history, thus fulfilling the criteria for cOP.

Vertebral morphometry was performed on 95 children: five patients showed vertebral fractures, of whom four were asymptomatic. **Table 6** provides detailed information about the vertebral fractures identified through morphometry in these patients, including fracture types and associated diagnoses.

**Table 6:** Vertebral fractures by morphometry.

N° Fractures	Fracture Type	Gender	Age (years)	Diagnosis	Other Fractures	LBMca
5	4 wedge (2 mild, 2 moderate) 1 severe biconcave	Female	13	Lymphoma	No	No
1	T7 mild wedge	Female	15	Polyartheritis Nodosa	No	No
1	T7 mild wedge	Male	11	Juvenile Idiopathic Arthritis	Yes	Yes
1	T8 moderate wedge	Female	11	Acute Lymphoblastic Leukemia + Hypopituitarism	Yes	Yes
1	T7 mild biconcave	Female	11	Cow’s Milk Protein Allergy	No	No

LBMca, Low Bone Mass for chronological age.

### 3.4 Association of clinical risk factors and BMD value

The correlation between clinical risk factors and BMD was assessed. A statistically significant relationship between vertebral BMD and age (p<0.001), sex (p<0.001), presence of hypovitaminosis D (p<0.001), time since diagnosis (p<0.001) and Latin American ethnicity (p<0.001) was found with a combined correlation coefficient of 0.73. This indicates that these risk factors accounted for 73% of the observed variability in vertebral BMD.

There was also a statistically significant relationship between total body BMD and age (p<0.001), time since diagnosis (p<0.001) and sedentary behaviors (p<0.001), with a combined correlation coefficient of 0.81 (p<0.001) and a statistically significant relationship between total body BMD less head and age (p<0.001), time since diagnosis (p<0.001), level of physical activity (p<0.001), and Latin American ethnicity (p=0.031). In this context, Latin American ethnicity may reflect underlying sociocultural, environmental, or genetic factors that were not directly measured in this study.

There was a statistically significant positive correlation between the number of risk factors and BMD in the three major regions of interest, but this relationship lost statistical significance when stratified by age.

A stepwise backward elimination of variables was performed to select the best predictive linear regression model. Finally, a linear regression test adjusted by age was performed to evaluate the association of risk factors and BMD values in different regions (**Table 7**). Vertebral BMD had a positive adjusted association with male sex and hypovitaminosis D. Whole body BMD had a negative adjusted association with a sedentary lifestyle and a history of fracture. Total body less head BMD had a negative adjusted association with current steroid treatment, a sedentary lifestyle, and a history of fracture.

## 4 Discussion

The objective of this study was to identify patients at risk of presenting LBMca based on their risk factors, describe this population, and assess the prevalence of LBMca and cOP. Additionally, the study aimed to investigate the impact of each risk factor on BMD. Our findings offer novel insights into the prevalence and influence of various risk factors on BMD within a growing population at risk of LBMca in a real-world setting.

### 4.1 Risk factors in our population

The patients included in the study were initially identified based on having one risk factor for developing LBMca. Upon further evaluation, we found that more than one-third of these patients had two or more risk factors. Notably, over one-quarter had at least four risk factors, which had previously gone unnoticed. This finding underscores the need for greater vigilance in identifying and monitoring risk factors to prevent them from being overlooked.

A higher number of risk factors was observed in patients with hematologic diseases, which could be expected given their frequent comorbidities. Additionally, the number of risk factors increased with age. However, these subgroup patterns are preliminary and should be viewed as hypothesis-generating, since subgroup sample sizes were small, particularly in the hematologic subgroup (n=7). To our knowledge, no other published studies of similar cohorts have described this observation, and further research is needed to validate these findings.

The most prevalent risk factor in this study was low calcium intake, which was found in 84.5% of the total cohort. Previous studies had observed a decrease in calcium intake in young, healthy individuals who were completing the transition to adulthood (23). A high percentage of inadequate calcium intake favors the presence of additional risk factors.

The relatively low fracture history (13/103) despite the prevalence of risk factors is not due to under-reporting, as our methodology included thorough examination of clinical histories for both personal and familial fracture incidents. Our approach was strengthened by implementing DXA morphometry assessment specifically to identify vertebral fractures that might otherwise go undetected. This proactive screening revealed a higher prevalence of asymptomatic vertebral fractures that initially anticipated, underscoring the importance of systematic radiological assessment in this population beyond reliance on reported symptoms or clinical history alone.

### 4.2 Diagnosis groups and BMD differences

When assessing whether there were differences in BMD according to the diagnosis group, it was observed that hematologic diseases along with digestive diseases and nephropathies were the groups with the lowest BMD. Although prior literature supports decreased BMD in relation to these conditions (24–30), our results are not conclusive due to small sample sizes within each subgroup. Nephrotic syndrome is one of the most widely studied diseases (29, 31, 32), and prior evidence illustrates that up to 25% of affected children present Z-scores lower than expected when compared to an average population one year after diagnosis (33).

In our study, hematologic diseases showed the highest proportion of LBMca (60%), followed by Juvenile Idiopathic Arthritis (JIA) and digestive diseases. While these trends are consistent with prior studies, they should be interpreted as exploratory given the limited subgroup sizes and thus require confirmation in larger, prospective cohorts.

### 4.3 Impact of risk factors on BMD

We observed that the main risk factors related to BMD were age and sedentary lifestyle, which accounted for 81.9% of the variability in total body BMD. For total body less head BMD, these two factors, along with Latin American ethnicity, likely serving as a proxy for broader unmeasured sociocultural, environmental, or genetic factors, explained 82.5% of the variability. Regarding vertebral BMD, a sedentary lifestyle was not found to be associated with variability in BMD. However, hypovitaminosis D, along with age, sex, and ethnicity, were found to be statistically significant related factors. The positive association between hypovitaminosis D and vertebral BMD may be influenced by several factors, including seasonal fluctuations in sunlight exposure (34, 35), the patient’s ethnicity (36), genetic polymorphisms (34, 35, 37), underlying diseases (35), vitamin D supplementation history, and other potential confounders (37). In particular, ethnic differences in vitamin D metabolism and seasonal variations in solar exposure could contribute to variability in vitamin D levels.

Moreover, prior studies, including ours, have described lower vitamin D concentrations in individuals with higher body weight (36). In the pediatric population, weight gain is often related to a higher BMD (38–40), a trend also described in our sample. Therefore, the relationship between Vitamin D and body weight may exert a further confounding effect in the interpretation of hypovitaminosis D and its effects on BMD, particularly in a growing population experiencing both weight gain and increasing BMD over time.

Additionally, disease severity in children with chronic diseases could play a critical role in both vitamin D metabolism and BMD. Similarly, vitamin D supplementation may modify the observed association between hypovitaminosis D and BMD, as those receiving supplementation could exhibit different bone health outcomes. Given these considerations, we interpret the positive association between hypovitaminosis and vertebral BMD with caution. Further sensitivity analyses, including adjustments for these potential confounders, are warranted to validate this finding. The complexity of the relationship between vitamin D, weight, and BMD in pediatric populations underscores the need for additional studies to clarify these associations.

Interestingly, whole body less head BMD had a negative adjusted association with current steroid treatment, but vertebral BMD did not, even though trabecular bone would be expected to be more sensitive to glucocorticoids. Previous histomorphometric studies describe that the use of corticosteroids in the pediatric population is associated with a decrease in trabecular thickness and the thickness of the osteoid material, together with an increase in the space between trabeculae, but these children are also reported as presenting heterogeneous and hypermineralized mineralization (41). The authors postulate that this heterogeneous hypermineralization, may partially explain why whole body less head may be more sensitive for evaluating the bone effects of glucocorticoids, since the evaluated area is larger and probably less sensitive to heterogeneity in mineralization.

It can be deduced from these data that sedentary lifestyle is the modifiable risk factor with the greatest impact on BMD in pediatric age. However, due to the cross-sectional design of this study, causality cannot be inferred, thus longitudinal cohort studies are needed to better understand temporal associations. In this study, a link between a decrease in the level of physical activity and increasing age was observed, and this observation has also been described in a comparable healthy population (17, 42). This decrease in the level of physical activity is a growing concern, and is also important in both healthy adolescents and adolescents suffering from chronic diseases, especially because of the well-known benefits of physical activity for both groups (43–48). In this respect, there are studies in which a lower level of physical activity is described in children and adolescents with JIA despite an adequate management of the disease (49). The same lower level was observed in children suffering from hematologic diseases 10 months after receiving their last treatment (50), as well as in children with chronic nephropathies (51), and in children with systemic autoimmune diseases (52), among others. Our study found a lower level of physical activity in children with hematologic diseases, followed by nephropathies, vasculitis, and autoinflammatory diseases.

There was no statistically significant association between the number of risk factors and total body, total body less head, and vertebral BMD measurements stratified by age. However, there was a clear trend of patients with a higher number of risk factors presenting a lower BMD. Although this study did not find a statistically significant relationship between any risk factors, or a combination of them, and LBMca, the authors believe it is important to consider them when evaluating children at risk of fractures.

A major limitation of our study is the inability to calculate Z-scores for the Total Body Less Head projections due to technical constraints of outdated DXA software, which undermines the standardization and comparability of these measurements. To mitigate this issue, we prioritized the use of raw BMD values alongside the available Z-scores, enhancing transparency and emphasizing their role in preserving the interpretability of our findings. While we believe the raw BMD values offer valuable clinical insights, they may not be as easily interpretable as Z-scores. These limitations should be taken into account when interpreting our findings, and further research with updated DXA software and standardized protocols is warranted to validate and expand upon our results.

The study’s cross-sectional design also represents a limitation, as it restricts our ability to draw causal inferences. Although we observed associations between sedentary lifestyle, hematological diseases, and BMD, these findings should be interpreted with caution. Further longitudinal studies are necessary to establish the temporal relationship between clinical risk factors and the development of LBMca or cOP over time. Moreover, the relatively small sample size (n=103) limits the statistical power of subgroup analyses. As such, all subgroup findings should be interpreted as exploratory.

Regarding data collection, another limitation concerns potential measurement bias in dietary calcium intake and physical activity levels. While the INDICAD questionnaire to estimate calcium intake is practical, it remains subject to recall bias inherent in self-reported dietary assessments. Likewise, physical activity questionnaires are not validated for children under three years of age, leading to incomplete data from this subgroup.

Despite these limitations, our study offers valuable insights. To our knowledge, this is the first study to investigate the prevalence and relevance contribution of each risk factor on BMD in a real setting of a pediatric growing population at risk of LBMca. While the current data provide an important foundation, future research should aim for larger, longitudinal designs.

**Figure 2:**
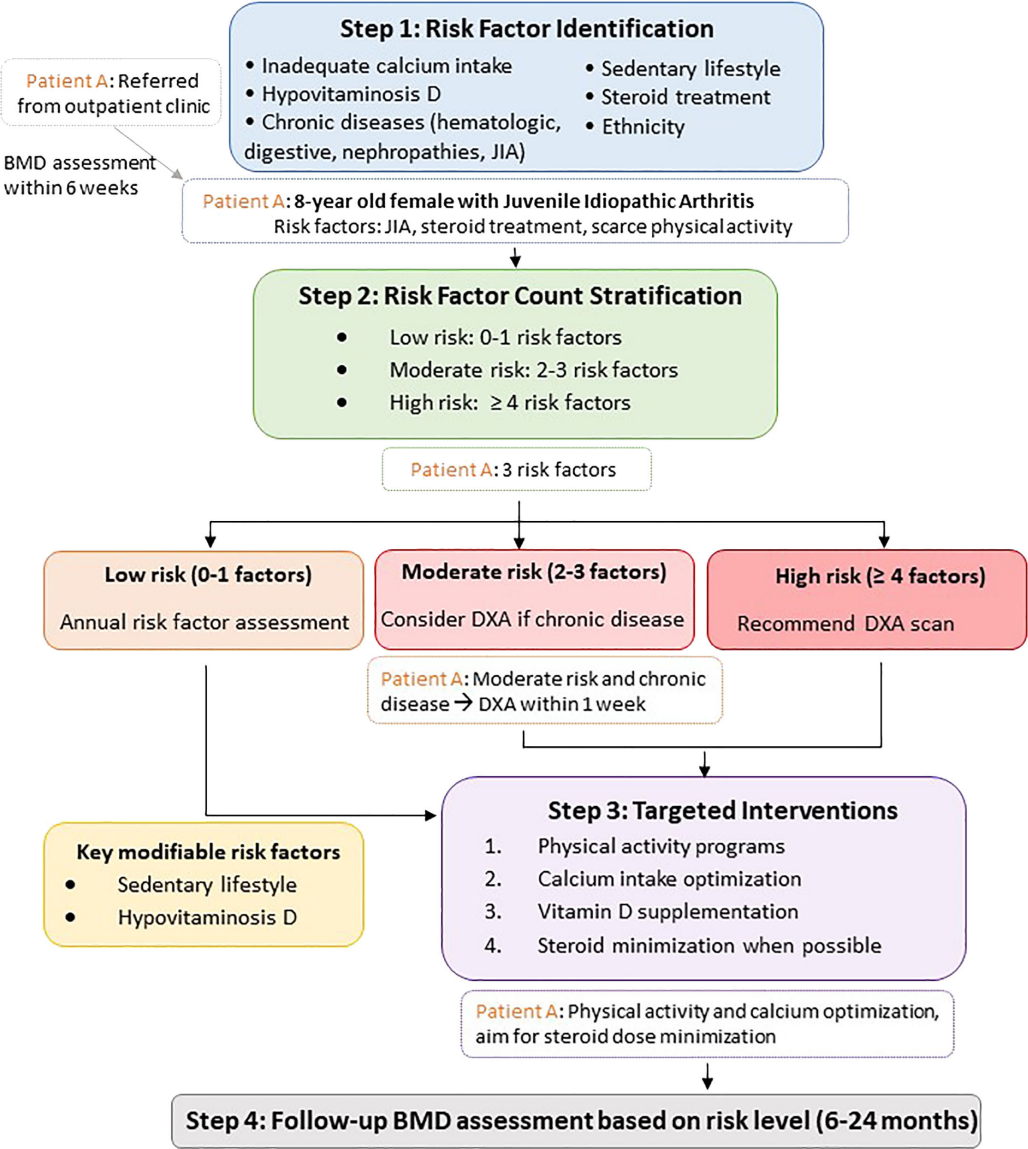
Screening Algorithm for Pediatric LBMca Risk. *A representative patient pathway has been added to illustrate the clinical decision process. LBMca, Low Bone Mass for chronological age; BMD, Bone Mineral Density; JIA, juvenile idiopathic arthritis; cOP, Childhood Osteoporosis.

Based on our findings regarding risk factor prevalence and their impact on BMD, we propose a screening algorithm for patients at risk of LBMca (**Figure 2**). This algorithm emphasizes the identification of risk factors and provides a structured approach to risk stratification and monitoring. The role of modifiable factors, especially physical activity, is further emphasized as a key public health message. Implementation of such a screening protocol in clinical practice could facilitate timely intervention in pediatric populations with chronic diseases, potentially preventing long-term bone health complications. While our cross-sectional data cannot establish causality, this screening framework offers a practical application that could inform clinical guidelines for pediatric bone health assessment.

**Table 7:** Best predictive regression model adjusted by age.

Variable	Age-adjusted regression Coefficient (95%CI)							
	Vertebral BMD	p	Total Body BMD	p	Total Body Less Head BMD	p	Trabecular Bone Score (TBS)	p
Sex (male)	+0.059 (0.041, 0.159)	**0.00**	+0.10 (0.041, 0.159)	0.51	+0.004 (-0.055, 0.063)	0.81	-34.04 (-63.44, -4.64)	0.08
Calcium intake under DRA	+0.037 (-0.022, 0.096)	0.22	+0.041 (-0.018, 0.100)	0.07	+0.004 (-0.055, 0.063)	0.06	-41.62 (-71.02, -12.22)	0.19
Comorbid disease	-0.043 (-0.102, 0.016)	0.67	+0.026 (-0.033, 0.085)	0.74	-0.038 (-0.097, 0.021)	0.64	-21.16 (-50.56, 8.24)	0.81
>1 comorbid disease	+0.046 (-0.013, 0.105)	0.33	+0.047 (-0.012, 0.106)	0.23	+0.043 (-0.016, 0.102)	0.3	-6.87 (-36.27, 22.53)	0.87
IS treatment	+0.009 (-0.050, 0.068)	0.68	-0.017 (-0.076, 0.042)	0.31	-0.02 (-0.079, 0.039)	0.26	-18.89 (-48.29, 10.51)	0.35
Steroid treatment	-0.016 (-0.075, 0.043)	0.53	-0.033 (-0.092, 0.026)	0.09	-0.047 (-0.106, 0.012)	**0.02**	-25.16 (-54.56, 4.24)	0.29
Previous steroid treatment	+0.05 (0.009, 0.109)	0.06	+0.039 (-0.020, 0.098)	0.05	+0.039 (-0.020, 0.098)	0.07	+3.10 (-26.30, 32.50)	0.90
Accumulated steroid doses	-<0.001 (-0.059, 0.059)	0.74	-0.000 (-0.059, 0.059)	0.73	-<0.001 (-0.059, 0.059)	0.72	-0.003 (-29.43, 29.42)	0.25
Duration of steroid treatment	+<0.001 (-0.059, 0.059)	0.57	+0.000 (-0.059, 0.059)	0.66	+<0.001 (-0.059, 0.059)	0.90	+0.19 (-29.21, 29.59)	0.66
Hypovitaminosis D	+0.083 (0.004, 0.162)	**0.03**	+0.049 (-0.010, 0.108)	0.09	+0.054 (-0.005, 0.113)	0.08	+13.91 (-15.49, 43.31)	0.67
Sedentary lifestyle	-0.052 (-0.111, 0.007)	0.09	-0.083 (-0.142,-0.024)	**0.00**	-0.091 (-0.150,-0.032)	**0.00**	-34.1 (-63.50, -4.70)	0.22
Fracture history	-0.060 (-0.119,-0.001)	0.05	-0.048 (-0.107, 0.011)	**0.04**	-0.055 (-0.114, 0.004)	**0.03**	-25.87 (-55.27, 3.53)	0.34
Hypercalciuria	-0.005 (-0.064, 0.054)	0.94	+<0.001 (-0.059, 0.059)	1.00	+0.034 (-0.025, 0.093)	0.48	+17.49 (-11.91, 46.89)	0.74
Proteinuria	+0.016 (-0.043, 0.075)	0.56	+0.023 (-0.036, 0.082)	0.27	+0.032 (-0.027, 0.091)	0.14	-23.18 (-52.58, 6.22)	0.37
Calcium supplement intake	-0.068 (-0.127,-0.009)	0.05	-0.046 (-0.105, 0.013)	0.08	0.038 (-0.021, 0.097)	0.17	-27.17 (-56.57, 2.23)	0.40
Vit D supplement intake	+0.009 (-0.050, 0.068)	0.76	-0.007 (-0.066, 0.052)	0.73	-0.001 (-0.060, 0.058)	0.95	+0.73 (-28.67, 30.13)	0.98
Caucasian	+0.014 (-0.045, 0.073)	0.60	+0.034 (-0.025, 0.093)	0.8	+0.036 (-0.023, 0.095)	0.08	+54.53 (25.13, 83.93)	0.02
Latin American	-0.047 (-0.106, 0.012)	0.17	-0.040 (-0.099, 0.019)	0.12	-0.067 (-0.126,-0.008)	0.01	-92.51 (-121.91, -63.11)	0.01

BMD, Bone Mineral Density; IS, immunosuppressant.

Emphases (bold text) are used for "p-values" reaching statistical significance.

## 5 Conclusions

In our cohort, LBMca and cOP prevalence in children with risk factors was up to 10.5% and 4.85%, respectively. Children at risk of LBMca/cOP typically presented with two or more risk factors identified, including age, sex, sedentary lifestyle, ethnicity, and hypovitaminosis D. While associations between these factors and bone health were observed, causality remains uncertain due to the cross-sectional nature of the study. Our findings support integrating routine DXA morphometry screening into clinical pathways for pediatric rheumatology and endocrinology, especially for at-risk children, as this revealed asymptomatic vertebral fractures that would have otherwise gone undetected. To fully understand the impact of these factors on the development of bone health and their potential long-term consequences, larger prospective studies are required.

## Data Availability

The original contributions presented in the study are included in the article. Further inquiries can be directed to the corresponding authors.
